# Molecular understanding and clinical outcomes of CAR T cell therapy in the treatment of urological tumors

**DOI:** 10.1038/s41419-024-06734-2

**Published:** 2024-05-24

**Authors:** Gong Zhang, Yuan Wang, Shiyang Lu, Fengzhu Ding, Xia Wang, Chunming Zhu, Yibing Wang, Kefeng Wang

**Affiliations:** 1 Department of Urology, Shengjing Hospital of China Medical University, Shenyang, 110004 China; 2 Department of General Surgery, Shengjing Hospital of China Medical University, Shenyang, 110004 China; 3 Department of Nursing, Shengjing Hospital of China Medical University, Shenyang, 110004 China; 4 Department of Family Medicine, Shengjing Hospital of China Medical University, Shenyang, 110004 China

**Keywords:** Urological cancer, Immunotherapy

## Abstract

Chimeric antigen receptor engineered T (CAR T) cell therapy has developed rapidly in recent years, leading to profound developments in oncology, especially for hematologic malignancies. However, given the pressure of immunosuppressive tumor microenvironments, antigen escape, and diverse other factors, its application in solid tumors is less developed. Urinary system tumors are relatively common, accounting for approximately 24% of all new cancers in the United States. CAR T cells have great potential for urinary system tumors. This review summarizes the latest developments of CAR T cell therapy in urinary system tumors, including kidney cancer, bladder cancer, and prostate cancer, and also outlines the various CAR T cell generations and their pathways and targets that have been developed thus far. Finally, the current advantages, problems, and side effects of CAR T cell therapy are discussed in depth, and potential future developments are proposed in view of current shortcomings.

## Facts

- The latest progress of CAR T cell therapy in urinary system tumors.
- The advantages, problems, and side effects of CAR T cell therapy in urinary system tumors.
- Mechanism and clinical application of novel immunotherapies in urinary system tumors.
- The various studies of CAR T generations and their pathways and targets in urinary system tumors.

## Open questions

- How to ensure the specificity and personalization of CAR T cell therapy in patients with urinary system tumors?
- What are the possible future directions of CAR T cell therapy in urinary system tumors?
- How can the safety and efficacy of CAR T cell therapy be improved through more extensive clinical trials and long-term follow-up?

## Introduction

According to statistics reported in the 2024 edition of CA, a Cancer Journal for Clinicians, urinary system tumors are among the most common malignancies in the United States, accounting for ~24% of all new cancers [1], with a rising incidence recently. In addition to surgery, some traditional treatment options, such as chemotherapy for urothelial carcinoma (UC) or androgen deprivation therapy (ADT) for prostate cancer (PCa), result in suboptimal clinical outcomes. For example, even after radical treatment of PCa, recurrence remains possible, especially for high-risk locally progressive PCa, which has a biochemical recurrence rate of up to 50% within 3 years after surgery [2]. As a result, immunotherapy approaches have gained increased attention in research.

At present, immunotherapy has been applied to multiple types of urinary tumors. In high-risk, non-muscle invasive bladder cancer (NMIBC), Bacille Calmette-Guérin (BCG) has been used to activate the mucosal immune system locally, stimulating an inflammatory response, which reduces recurrence risk [3]. In renal cell carcinoma (RCC), systemic interleukin-2 (IL-2) treatment activates the immune system, mainly by stimulating natural killer (NK) cells and T cells to kill tumor cells [4]. In addition, immune checkpoint inhibitors (ICIs), such as those impacting programmed death 1 (PD-1) and programmed death-ligand 1 (PD-L1), have been used increasingly to treat urinary system tumors [5].

More recently, chimeric antigen receptor engineered T cell (CAR T) therapy, a novel genetically based immunotherapy method, has been applied increasingly in malignancy [6]. CAR T cell therapy has achieved impressive results in treating a variety of hematologic malignancies, including lymphoma, leukemia, and multiple myeloma [7]. The availability of tumor-associated antigens in urinary system tumors, such as prostate stem cell antigen (PSCA), prostate specific membrane antigen (PSMA), and epidermal growth factor receptor (EGFR), makes CAR T cell therapy possible for urinary system tumors. However, the immunosuppressive tumor microenvironment (TME), antigen escape, tumor heterogeneity, and the diminished proliferation capacity, insufficient migration ability, and short survival time of CAR T cells, limit their impact for solid tumors [8].

In this manuscript, we systematically review the research progress, potential value, and difficulties of utilizing CAR T cells to treat urinary system tumors, to provide novel approaches for urinary system tumor treatment.

## CAR T cell therapy in tumor immunotherapy

Immunotherapy recently has developed rapidly for both cancer and regenerative medicine. In oncology, multiple clinical trials of autologous engineered T cells are underway, with the goal of improving anti-tumor immune responses, and this approach may change cancer treatment standards [9].

CAR T cell therapy is one type of adoptive cell therapy, with the major advantages of not being restricted by the major histocompatibility complex, bypassing antigen presentation, priming and activating T cells, which directly attack cancer cells, killing them [10]. CAR T cell therapy has proven efficacious for treating hematological malignancies, especially B-cell malignancies [11]. However, for solid tumors, such as UC, CAR T cell therapy requires further development before it becomes widespread.

**Fig. 1:**
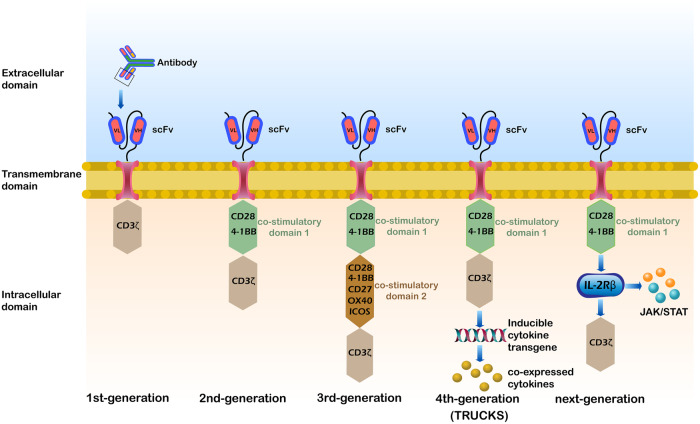
Structural comparison of different generations of CAR T. The first-generation of CAR T had only CD3ζ chains. The second-generation of CAR T added co-stimulatory domain 1 (CD28 or 4-1BB) on the basis of the first-generation. The third-generation of CAR T added co-stimulatory domain 2 (CD28, 4-1BB, CD27, OX40, and ICOS) on the basis of the second-generation. The fourth-generation of CAR T added co-expressed cytokines on the basis of the second-generation. The next-generation of CAR T was also based on the second-generation, with the addition of co-stimulatory domains for the activation of other signaling pathways, such as IL-2Rβ-JAK/STAT pathway.

The engineered receptors of CAR T cells consist of three major components: an extracellular domain specific for tumor antigens [single chain fragment variable (scFv) fragment], a transmembrane domain, and an intracellular signaling domain that activates T lymphocyte responses (including costimulatory molecules) [12] (Fig. 1). Initial in vivo testing of the first-generation of CAR T cells, which had basic architecture, revealed poor activation and persistence of the engineered T cells [13]. Further studies revealed that full T cell activation requires the first signal be transmitted upon binding of the extracellular antigen binding domain to the antigen, as well as a second signal transmitted by the binding of costimulatory molecule receptors to their ligands, but these ligands are usually not expressed on the tumor cell surface. Enhanced CAR T cells have come from optimizing T cell proliferation and tumor kill by including intracellular costimulatory molecules in the receptor. The design structure of the first-generation of CAR T cells was relatively simple, with the CD3ζ costimulatory domain as the only intracellular domain included, with none of the costimulatory signals necessary to fully activate the CAR T cells, and their effect in clinical trials was suboptimal. Based on the design of the first generation of CAR T cells, the costimulatory domains CD28 or 4-1BB were introduced in the second generation of CAR T cells, significantly improving their immune activation and durability [14, 15]. CD28 and 4-1BB exhibited different anti-tumor properties. CD28-activated T cells had a strong transient mortality, while 4-1BB-activated T cells had better anti-tumor persistence [16].

Expanding on these results, the third-generation of CAR T cell receptors contained two co-stimulatory domains, usually CD28 and 4-1BB, but also sometimes CD27, OX40, or ICOS [17–20]. In preclinical models, third-generation anti-CD19 CAR T cells with both CD28 and 4-1BB co-stimulatory domains provided better therapeutic outcomes than did second-generation CAR T cells. These cells showed balanced tumor-killing and increased persistence, with an elevated CD8/CD4 ratio and decreased exhaustion [21]. However, there are significant differences in cytokine production and anti-tumor activity in these third-generation of CAR T cells compared with the second-generation. This difference may be due to impaired mitochondrial function caused by over-stimulation of CAR T cells, leading to CAR T cell exhaustion [22].

Based on these observations, the fourth-generation of CAR T cells added intracellular co-expressed cytokines (IL-7, IL-12, IL-18, IL-21, CCL19, et al.) to positively regulate CAR T cells [23–27]. These have an additional inducible domain responsible for transgenic cytokine release upon CD3ζ-containing CARs engaging with their specific target. The activation of these receptors leads to secretion of the cytokine to improve CAR T cell persistence and antitumor effects [28, 29]. For example, IL-12 is eventually produced and released in target-initiated CD3ζ-containing CAR T cells. IL-12 promotes T cell activation, modulates the immune and vascular tumor environment, and recruits additional immune cells to attack tumor cells not recognized by the CAR T cells [30, 31]. Therefore, the fourth-generation of CAR T cells are also termed T cells redirected for universal cytokine-mediated killing (TRUCKs).

The fifth-generation of CAR T cells, also based on the second-generation, have added an additional intracellular IL-2 receptor domain that enables antigen-dependent activation of the JAK/STAT pathway [32, 33]. This induces the CAR T cells to produce memory T cells, facilitating a more durable long-term response. The fifth-generation of CAR T cells also have additional intracellular domains with truncated cytokine receptor fragments, such as IL-2R chain fragments, containing transcription factor binding motifs. Therefore, these secreted signals not only drive CAR T cell activity persistence and memory T cell generation, but also reactivate and stimulate the immune system [32] (Fig. 1). Naeem et al. [34] also tried to circumvent other challenges with these cells by using clustered regularly interspaced short palindromic repeats (CRISPR) genome editing technology to inhibit dominant negative receptors (PD-1, TGF-β, and B2M), reduce the risk of cytokine release syndrome (CRS), and regulate CAR T cell function in the TME.

## CAR T cell therapy in urologic neoplasms

Given the impressive therapeutic effects of CAR T cells for multiple hematological malignancies [7], investigations related to urinary system tumors recently have been conducted with increasing frequency. CAR T cell therapy is made possible by the expression of tumor-associated antigens in urinary system tumors, permitting a novel approach for the treatment of urinary system tumors, including RCC, PCa, and bladder cancer (BCa) (Table 1).

**Table 1:** CAR T cell therapy in urinary system tumors.

Tumors	Target antigens	Generic/specific antigens	Co-stimulatory molecules	Generations of CAR T	Characteristics of CAR T	Refs
RCC	CAIX	Generic	CD28	4th-generation	Secrete anti-PD-L1 antibodies at the tumor site to block T cell exhaustion.	[50]
	CD70	Generic	CD27	2nd-generation	Potent anti-tumor activity against RCC cell lines and patient-derived xenograft mouse models.	[51]
	c-Met	Generic	CD28, 4-1BB	3rd-generation	Induce marked infiltration of the CAR T cells into the tumor tissue and synergistically augment with axitinib.	[52]
	CAIX	Generic	4-1BB	2nd-generation	Synergistically augment with sunitinib.	[53]
BCa	PD-1	Generic	CD28	2nd-generation	May be able to target multiple types of solid tumors.	[60]
	MUC1	Generic	CD28	2nd-generation	It has been confirmed by preclinical studies in vitro.	[61]
	EGFR	Generic	CD28	2nd-generation	Combined with decitabine can enhance tumor specific killing.	[62]
PCa	PSMA	Specific	4-1BB	2nd-generation	Co-expression of dnTGF-βRII enhances the anti-tumor effect of PSMA-targeting CAR T cells.	[67]
	PSMA	Specific	CD28	2nd-generation	Significant synergistic enhancement with low doses of docetaxel.	[69]
	PSMA	Specific	4-1BB	4th-generation	IL-23mAb combined with PSMA CARs was more effective in eradicating PCa.	[70]
	PSCA	Generic	4-1BB	2nd-generation	4-1BB-containing CARs show superior T cells persistence and control of disease compared with CD28-containing CARs.	[71]
	STEAP1	Specific	4-1BB	2nd-generation	Enhance anti-tumor efficacy by combining CBD-IL12 fusion protein.	[74]
	B7-H3	Generic	CD28	2nd-generation	Produce favorable anti-tumor effects on PCSCs treated by fractionated irradiation.	[75]

### CAR T cell therapy in RCC

In the United States, RCC is twice as common in men as in women, with ~81,610 new cases and 14,390 deaths expected in 2024 [1]. The incidence of RCC has recently stabilized, rather than improving, despite advances in surgical treatment, such as robot-assisted surgical treatment, suggesting the need for new treatments [35, 36]. Since RCC does not respond to radiotherapy and chemotherapy, IL-2 and interferon-α are the traditional non-specific immunotherapies used [37, 38], but multiple studies have questioned their effectiveness [39]. More recently, for advanced RCC, the current standard first-line immunotherapies include dual ICIs and ICIs combined with vascular endothelial growth factor receptor tyrosine kinase inhibitors [40]. Therefore, new immunotherapy has become an area of active interest.

**Fig. 2:**
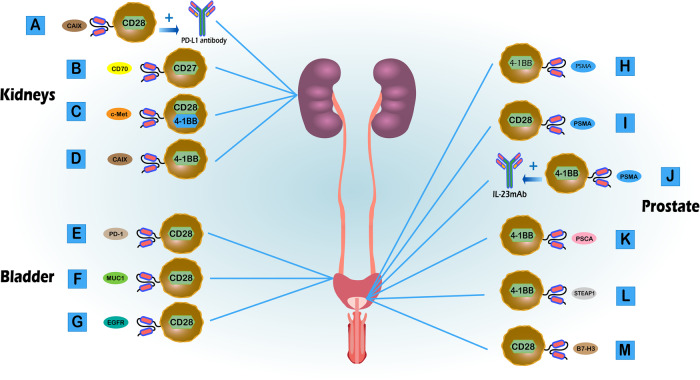
Application of different types of CAR T cell therapy in urinary system tumors. **A** The fourth-generation of CAR T (CD28) targeted CAIX to secrete anti-PD-L1 antibody to block T cells exhaustion in RCC. **B** The second-generation of CAR T (CD27) targeted CD70 in RCC. **C** The third-generation of CAR T (CD28 and 4-1BB) targeted c-Met in RCC. **D** The second-generation of CAR T (4-1BB) targeted CAIX in RCC. **E** The second-generation of CAR T (CD28) targeted PD-1 in BCa. **F** The second-generation of CAR T (CD28) targeted MUC1 in BCa. **G** The second-generation of CAR T (CD28) targeted EGFR in BCa. **H** The second-generation of CAR T (4-1BB) targeted PSMA in PCa. **I** The second-generation of CAR T (CD28) targeted PSMA in PCa. **J** The fourth-generation of CAR T (IL-23mAb-PSMA-CARs) was more effective in PCa. **K** The second-generation of CAR T (4-1BB) targeted PSCA in PCa. (**L**). The second-generation of CAR T (4-1BB) targeted STEAP1 in PCa. **M** The second-generation of CAR T (CD28) targeted B7-H3 in PCa.

CAR T cell therapy has been studied in RCC. Suarez et al. [41] developed CAR T cells targeting human carbonic anhydrase IX (CAIX) to secrete human anti-PD-L1 antibodies at the tumor sites (Fig. 2A). In a humanized mouse model of RCC, tumor suppression was evident after treatment with CAIX-CAR-T cells. Panowski et al. generated a panel of anti-CD70 scFv-based CAR T cells [42]. In their study, CD70 CAR T cells showed potent anti-tumor activity against RCC cell lines and in patient-derived mouse xenograft models (Fig. 2B). In addition, Mori et al. [43] found that cellular-mesenchymal epithelial transition factor (c-Met) could be used as a therapeutic target for RCC, creating anti-human c-Met targeted CAR T cells (Fig. 2C) and studying their anti-tumor effects in an orthotopic mouse model. They found that anti-c-Met CAR T cells could infiltrate tumor tissues and inhibit tumor growth.

Multiple studies have found that CAR T cells combined with targeted drugs may have an improved anti-tumor effect compared with CAR T cells alone. Li et al. [44] constructed second-generation CAR T cells that recognize the human RCC-specific antigen, CAIX (Fig. 2D). In their investigation, the combination of CAIX-CAR-T and sunitinib had a synergistic effect on lung metastasis in a mouse model of human RCC. The CAIX-CAR-T cells in the combined treatment group had increased proliferation and invasion compared to that observed in the single treatment group. Mori et al. [43] had similar results. After the construction of c-Met-specific CAR T cells, they studied these in combination with axitinib, finding that axitinib synergistically enhanced anti-tumor CAR T cell responses. These results suggest that the combination of CAR T cells with targeted agents may also be effective for solid tumor therapy.

### CAR T cell therapy in BCa

In the United States, BCa, with ~83,190 new cases and 16,840 deaths expected in 2024, is more common in men [1], and BCa treatment is mainly based on risk classification. With the standardization of treatment, BCa prognosis has significantly improved recently. For example, NMIBC has a 5-year survival rate of up to 90%. However, outcomes for metastatic BCa (mBCa) remain particularly poor, with a 5-year survival rate of <15% [45, 46], with a median survival for various chemotherapy regimens of only 13–15 months [47, 48]. While ICIs have been considered an optional first-line treatment for patients who are not candidates for chemotherapy, the results remain unsatisfactory [49], highlighting the need for new immunotherapies. BCa expresses multiple tumor-associated antigens, suggesting favorable conditions for CAR T cell therapy.

Many pan-cancer tumor targets, including human epidermal growth factor receptor 2 (HER2), mucin 1 (MUC1), and EGFR, are also expressed at high levels in UCs and may serve as therapeutic targets [50, 51]. One current ongoing clinical trial targets HER2 (NCT03740256). However, this is a basket trial, and BCa is just one of several malignancies included. We look forward to learning the specific results in BCa. Parriott et al. [52] developed CAR T cells targeting PD-1, finding an anti-tumor effect for CHPD1-expressing T cells in a variety of isogenic mouse solid cancer models, including BCa (Fig. 2E). Yu et al. [53] produced second-generation CAR T cells targeting MUC1 in vitro (Fig. 2F), finding a significant, specific cytotoxic effect of MUC1-CAR-T on MUC1-positive tumors such as BCa, further validating the feasibility of using MUC1-CAR-T cells to treat BCa. In addition, Grunewald et al. [54] used decitabine in combination with EGFR and CD44V6-specific CAR T cells for anti-BCa studies (Fig. 2G), finding that the combination was an attractive therapeutic approach to enhance tumor-specific killing in BCa.

### CAR T cell therapy in PCa

With an estimated 300,000 new cases and 35,250 deaths anticipated in the United States in 2024, PCa is the most common malignancy in men and the second leading cause of cancer mortality [1]. The 5-year survival rate of PCa reaches 98%, but in metastatic PCa (mPCa), survival drops to 30% [55]. After years of experience, PCa treatment has become standardized. PCa growth and development depend on androgens, so ADT is the cornerstone of PCa treatment. Standard treatment for localized PCa includes surgery, radiation, and active surveillance [56]. However, the poor prognosis of patients with mPCa, especially castration-resistant PCa (CRPC), remains concerning. Diverse treatment options exist for advanced PCa, including taxane chemotherapy, Sipuleucel-T, androgen receptor (AR) pathway inhibitors, and ICIs [57]. PCa is often described as a “cold” tumor because of its limited sensitivity to ICIs [58]. The non-response to ICIs spurs interest in novel immunotherapy methods, such as CAR T cell therapy.

A variety of tumor specific antigens are expressed in PCa. In addition to the well-known PSA, there are PSMA and PSCA, which may serve as targets for CAR T cell therapy. PSMA is currently the most studied target for CAR T cell therapy in PCa. For example, Shanghai Changzheng Hospital has just registered a single-center, single-arm, open-label, investigator-initiated clinical trial evaluating the safety and efficacy of enhanced autologous PSMA-specific CAR T cells to treat refractory CRPC (NCT06228404). In addition, multiple preclinical and clinical investigations found that PSMA-specific CAR-T cells have an anti-PCa effect. Kloss et al. [59] enhanced the effect of CAR T cells targeting PSMA by co-expression of a dominant-negative TGF-βRII (dnTGF-βRII), which further increased lymphocyte proliferation and cytokine secretion, leading to killing of PCa cells (Fig. 2H). Subsequently, they conducted a phase I trial (NCT03089203) consisting of 18 patients, demonstrating that treatment with TGF-β-resistant CAR T cells is feasible and generally safe [60]. Alzubi et al. [61] developed a novel D7 single-chain antibody fragment-derived anti-PSMA CAR that showed promising activity both in vitro and in vivo (Fig. 2I). In addition, the combination of PSMA-CAR-T cells and non-ablative low-dose chemotherapy, such as low-dose docetaxel, controlled tumor growth. Wang et al. [62] constructed a group of IL-23 mAb-PSMA-CARs and found that IL-23 mAb combined with PSMA-specific CAR T cells were more effective in eradicating PCa than were PSMA CAR T cells alone (Fig. 2J).

In addition to PSMA, other targets have been evaluated. Priceman et al. [63] found the intracellular co-stimulatory signaling domain can determine the sensitivity of CAR T cells to tumor antigen expression. Inclusion of a 4-1BB intracellular co-stimulatory signal domain provided better tumor killing than did a PSCA-CAR containing a CD28 co-stimulatory signal domain (Fig. 2K). A phase Ib clinical trial (NCT05805371) of PSCA-targeting CAR T cells to treat metastatic CRPC is ongoing and will evaluate this question. The six transmembrane epithelial antigen of the prostate 1 (STEAP1) can be used as a target antigen for PCa therapy [64, 65]. Bhatia et al. [66] designed a STEAP1-targeted CAR T cell therapy with high antigen specificity (Fig. 2L), finding that STEAP1 CAR T cells showed reactivity at low antigen density, anti-tumor activity in mPCa models, and safety in human STEAP1 knock-in mouse models. Furthermore, the addition of tumor-localized IL-12 in the form of a collagen binding domain-IL-12 fusion protein enhanced the anti-tumor effect of the STEAP1 CAR T cells. Zhang et al. [67] reported that B7-H3 CAR T cells combined with fractionated irradiation (FIR) was more effective than FIR or CAR T cells alone for radioresistant PCa stem cells (PCSCs) (Fig. 2M).

## Advances and challenges

Multiple CAR T cell therapies have proven effective for treating even refractory B-cell malignancies, providing a new approach for cancer treatment and leading to progress for treatment of other malignancies. As mentioned earlier, tumor-associated antigens are crucial for the application of CAR T cell therapy. Tumor-related antigens commonly expressed in urinary system tumors and other solid tumors have shown good anti-tumor activity in preclinical or clinical studies, such as CAR T cells targeting PSMA, EGFR, MUC1, etc. Therefore, the use of CAR T cell therapy in urologic tumors may be more favorable, given the availability of target antigens expressed more frequently than is observed in other solid tumors.

In addition, CAR T cells still require a certain process before administration to patients, which creates challenges for delivering CAR T cell therapy in a timely fashion. However, urologic tumors progress relatively slowly compared to other malignancies, such as those of hematological, respiratory, and digestive system cancers, providing sufficient preparation time for specific and individualized CAR T cells. To shorten the time to CAR T cell infusion and reduce costs, researchers have evaluated using cells from healthy donors, that is, allogeneic T cells, to produce CAR T cells. However, two major problems exist when using allogeneic CAR T cells. First, allogeneic T cells may cause fatal graft-versus-host disease (GVHD). Second, these allogeneic T cells may be rapidly eliminated by the host immune system, limiting their effectiveness [68]. Fortunately, several studies have shown that the GVHD from CAR T cells is not a cause for concern. Studies of donor-derived CAR T cells therapy have exhibited that alloreactive T cells expressing CD28 co-stimulated CD19 CARs, thereby further significantly reducing the incidence of GVHD [69–71]. Further study is needed to confirm this observation.

CAR T cell therapy still has many shortcomings and faces serious challenges. Firstly, the current use of CAR T cells for solid tumors, including urological tumors, remains very limited. Secondly, from a safety perspective, the side effects continue to be significant, including CRS, immune effector cell-associated neurotoxicity syndrome (ICANS), and the resulting organ dysfunction [72].

Although CAR T cell therapy offers many advantages for urological tumors, its efficacy has not reached that of CAR T cells for hematological malignancies. In addition, the heterogeneity of the TME poses challenges for CAR T cells. The TME has multiple anti-tumor effects, most notably on T cell activity [73]. A large number of immunosuppressive cells, including regulatory T cells (Tregs), myeloid-derived suppressor cells, and tumor-associated macrophages, are usually present in the solid TME. Co-stimulatory molecules, such as CD28, may facilitate CAR T cell activation and survival at tumor sites [74, 75]. Various common cytokines also affect the efficacy of immunotherapy for urological tumors. For example, transforming growth factor-β (TGF-β), which is abundantly expressed in the TME of BCa and PCa, downregulates CD8^+^ T cell function and promotes Treg maturation [76]. Future investigations will examine how to overcome the inhibition of TGF-β on CAR T cells [77, 78].

Conversely, cytokines such as IL-2 and IL-12 can enhance the anti-tumor effects of CAR T cells [79]. IL-12, in particular, can alter the TME and prolong T cell survival, improving immunotherapy efficacy [30, 31, 80]. However, neither IL-2 nor IL-12 are normally present in the TME of urological tumors, but intravesical therapy with BCG after BCa surgery induces IL-2 and IL-12 in the TME, implying that CAR T cells expressing these cytokines may be advantageous for BCa [77]. Further studies evaluating the role of the TME on CAR T cells in solid tumors, especially urological tumors, are needed to improve the applicability of CAR T cells for these malignancies.

The most popular immunotherapy currently uses ICIs, which are also widely used in urological tumors and are coming to be accepted as a first-line treatment for advanced RCC and BCa [81, 82]. When ICIs prove ineffective, it is uncertain how impactful CAR T cell therapy, as another immunotherapy, will be. This question may be addressed by clinical trials that are currently underway. For example, the inclusion criteria of the clinical trials NCT05420519, targeting CD70, and NCT04969354, targeting CAIX, both had patients with advanced RCC who have failed first-line and second-line therapies (including ICIs). In addition, several preclinical and clinical trials in other tumors have shown that ICIs combined with CAR-T cell therapy can enhance anti-tumor activity, offering hope for this combination in urological tumors [83–85]. Ren et al. [86] also found that CAR T cells that use CRISPR to knock down the PD-1 encoding gene, PDCD1, exhibited potent anti-tumor activity both in vitro and in animal models, suggesting that such CAR T cells lacking this checkpoint receptor might be beneficial.

However, ICIs have limited utility for PCa, predominantly because of the previously-mentioned immunosuppressive TME. Beyond ongoing effort to enhance immune signals and remodel the immunosuppressive “cold” TME, the success of ICIs in combination with CAR T cells, especially PD-1 knockdown CAR T cells, provides novel approaches for improvement. Another possibility involves cancer-associated fibroblasts (CAFs), which are the most abundant component of the PCa TME [87]. If CAR T cells can be engineered to target the specific surface antigens of CAFs, might this inhibit the growth and infiltration of PCa? All of these ideas merit further exploration and validation.

Another challenge is CRS, a physiological inflammatory state triggered by the interaction of inflammatory cytokines and chemokines released by CAR T cells. These cytokines include granulocyte-macrophage colony-stimulating factor, interferon-γ, and tumor necrosis factor-α, and are released after T cells engage with their corresponding target antigens [88, 89]. CRS has been observed in many patients receiving CAR T cell therapy [72, 88, 89] and are the main side effect of CAR T cell therapy. Interestingly, CRS is not the result of CAR T cells themselves, but inflammatory cytokines released by macrophages in response to the activated CAR T cells, such as IL-1, IL-2, IL-6, and especially IL-6. When these inflammatory cytokines are released in large amounts, they cause an inflammatory response in the vascular endothelium, which further releases IL-6, leading to a positive feedback loop for CRS [89–92]. This effect disrupts vascular integrity in patients with CRS and leads to hemodynamic instability, capillary leakage, and consumptive coagulopathy [93, 94] (Supplementary Fig. S1). To predict CRS occurrence in high-risk patients in advance, Hay et al. [95] performed classification-tree modeling using data from 133 patients with CRS. They found that the monocyte chemoattractant protein 1 may be a useful prediction marker, and additional predictive markers with good specificity and sensitivity are being explored. Further clinical experience and practice are still needed for the management of CRS.

Neurotoxicity, another common acute side effect of CAR T cell therapy, has been observed in 64% of clinical trials [72]. ICANS may occur simultaneously with CRS, after resolution of CRS, or independent of CRS. Clinical manifestations of ICANS can include decreased consciousness, coma, seizures, motor weakness, and cerebral edema [93]. The pathophysiological mechanism of ICANS remains unclear, but hypotheses include the passive diffusion of cytokines during the transport of CAR T cells in the central nervous system, endothelial cell activation and blood-brain barrier destruction, and N-methyl-D-aspartate receptor agonists [93, 94, 96].

More extensive clinical trials and long-term follow-up are needed to improve the safety and efficacy of CAR T cells. Moreover, the high cost of CAR T cells are a barrier, highlighting the need to improve affordability for patients. The fourth and subsequent generations of CAR T cells are being designed to enhance their lethality, extend persistence after infusion, and enhance their ability to invade solid tumor tissues and regulate the TME [97–99]. The ongoing development of CAR T cells should also focus on optimizing target selection and construction methods, reducing toxicity, stabilizing the construction system, and improving invasiveness.

## Conclusion

In conclusion, numerous studies have demonstrated the potential utility of CAR T cells for urologic tumors. However, adoptive T cell immunotherapy still faces many challenges, including the viability of tumor cells, the complex regulatory mechanisms of the TME, and side effects such as CRS and ICANS. The ongoing optimization of CAR T cell therapy should focus on improving the role of CAR T cells in solid tumors. Cytokines that improve the structure of CAR T cells or certain pathways may achieve this goal. With the development and progress of a multiple areas of investigation, CAR T cells are poised to become increasingly useful for the treatment of solid tumors, including urinary system tumors.

### Supplementary information

Supplementary Figure S1

Supplementary Figure S1 legend
