# Supplementary figures and images for: Molecular understanding and clinical outcomes of CAR T cell therapy in the treatment of urological tumors
Source: Cell Death Dis. 2024 May 24;15(5):359. doi: 10.1038/s41419-024-06734-2 (PMC11126652; doi:10.1038/s41419-024-06734-2)

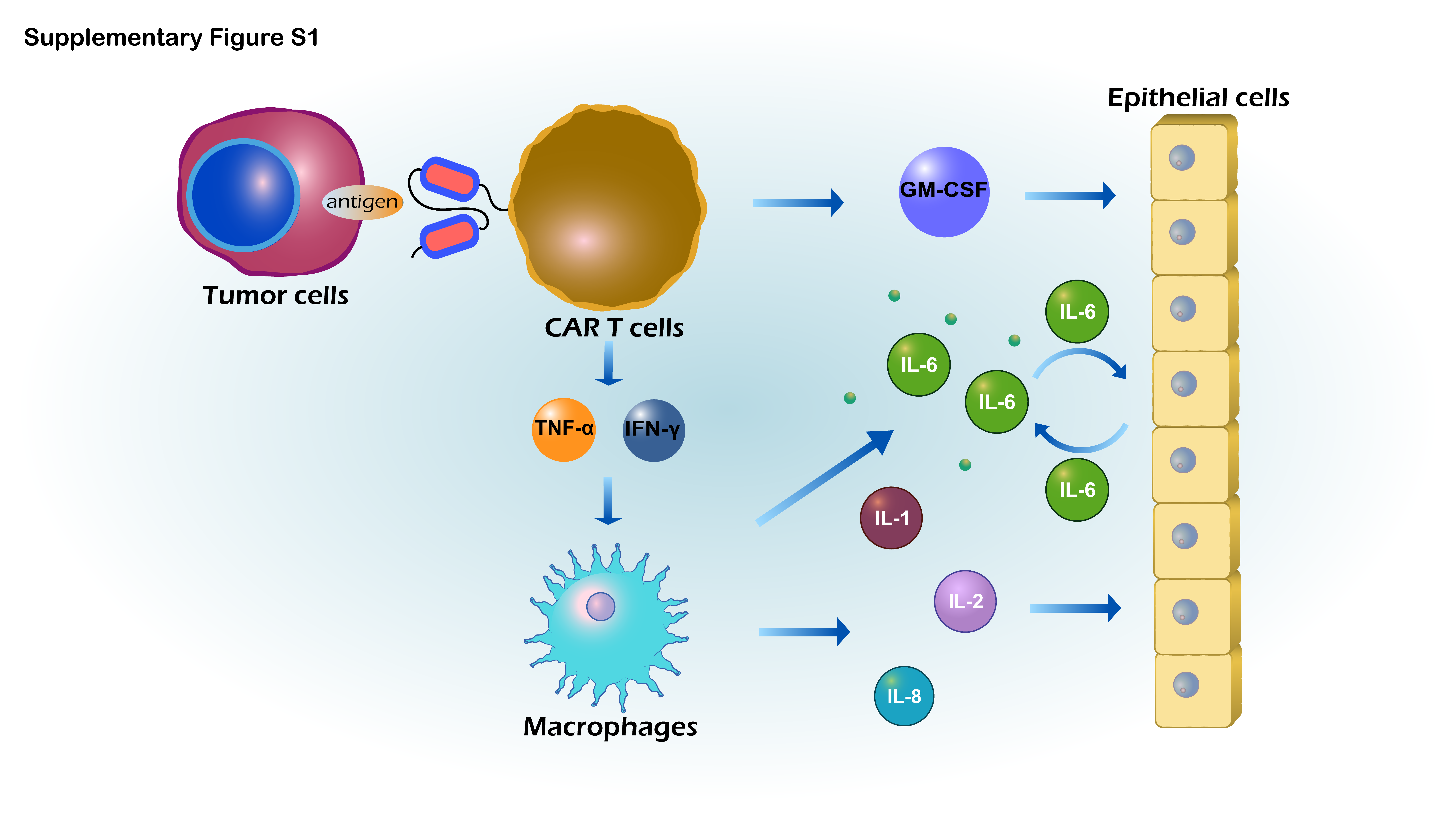

Supplement: Supplementary file 1 — Supplementary Figure S1 [file 41419_2024_6734_MOESM1_ESM.tif]
