# Supplementary material for: Molecular understanding and clinical outcomes of CAR T cell therapy in the treatment of urological tumors
Source: Cell Death Dis. 2024 May 24;15(5):359. doi: 10.1038/s41419-024-06734-2 (PMC11126652; doi:10.1038/s41419-024-06734-2)
Supplement: Supplementary file 2 — Supplementary Figure S1 legend [file 41419_2024_6734_MOESM2_ESM.docx]

**Supplementary Figure legends**

**Supplementary Figure S1. Current understanding in pathophysiology of CRS.**

CRS was a physiological inflammatory state triggered by the interaction of inflammatory cytokines and chemokines released by CAR T cells (such as GM-CSF, IFNγ, etc.) with the corresponding target antigens of tumor cells. The core of CRS was an inflammatory cytokine released by macrophages, mainly IL-6. They caused an inflammatory response in the vascular endothelium, which further releases IL-6, forming a positive feedback loop of CRS. Ultimately, it destroyed the vascular integrity of CRS patients, leading to problems such as capillary leakage.
